# The Mechanisms of Inclusive Leadership on Newcomers’ Proactive Socialization Behaviors—An Exploration Based on the Proactive Motivation Model

**DOI:** 10.3390/bs15010072

**Published:** 2025-01-16

**Authors:** Jingyi Shi, Long Ye, Junnan Ren

**Affiliations:** 1 School of Economics and Management, Beijing Jiaotong University, Beijing 100044, China; yelong@bjtu.edu.cn; 2 School of Education, Tianjin University, Tianjin 300072, China; nkrenjunnan@163.com

**Keywords:** inclusive leadership, proactive motivation model, individual power distance orientation, newcomers’ proactive behaviors

## Abstract

This study advances newcomers’ socialization research by identifying and investigating the antecedents of newcomers’ proactive behaviors, a perspective often overlooked within the current studies, which primarily focuses on the outcomes of such behaviors. Based on the proactive motivation model, our core hypothesis is that inclusive leadership allows newcomers to experience psychological changes in control beliefs (can-do motivation), state promotion focus (reason-to motivation), and positive affect (energized-to motivation), which are stimulated following proactive behaviors. We further consider an individual values variable—individual power distance orientation—as the boundary condition on the influence of inclusive leadership. Research was conducted on 353 newcomers with less than one year of work experience to test the above hypothesis. The results show that inclusive leadership positively and indirectly influences the newcomers’ proactive behaviors via state promotion focus and positive affect. However, the mediating effect of control beliefs was not significant. Furthermore, the positive association between inclusive leadership and two kinds of newcomer proactive motivations, and their accompanying indirect impacts on newcomers’ proactive behaviors, was proved stronger at lower levels of individual power distance orientation. Additionally, it has been discovered in a follow-up complementary study that the effect of control beliefs on newcomers’ proactive behaviors is transmitted through positive affect.

## 1. Introduction

Newcomers’ organizational socialization refers to the transition process of individuals from outsiders to insiders, which entails the acquisition of attitudes, behaviors, and knowledge that align with the organization’s values and norms (Bauer & Erdogan, 2011). In recent years, job transitions have become increasingly frequent and the stress resulting therefrom brings this topic back into the purview of academic research (Bauer et al., 2019) This necessitates that scholars identify more effective socialization strategies for newcomers within the current context. Originally, research on this topic focused on strategies to help newcomers adapt to the organization. Over time, however, studies found that newcomers are not passive recipients of the socialization process. Instead, they are driven by intrinsic motivations to adjust their attitudes and behaviors to match their environment or shift their role expectations to align with their personal needs. This is conceptualized as newcomers’ proactive behaviors, which are considered crucial in determining their successful integration into the organization (Kowsikka & James, 2019; Wu et al., 2023). According to Cooper-Thomas and Burke (2012), newcomers’ proactive behaviors such as information seeking, feedback seeking, relationship building, and positive framing actively promote their adaptation process.

Despite the recognized advantages, research lacks a comprehensive understanding of the causes of newcomers’ proactive behaviors. Given that proactivity is generally considered an intrinsic characteristic of individuals, studies have tended to overlook the factors influencing such behaviors. Instead, it primarily focuses on the potential socialization outcomes resulting from them (Nguyen et al., 2021). However, Parker et al. (2006) argue that the level of proactivity might fluctuate depending on the work environment, indicating that a newcomer may demonstrate a greater degree of proactive behaviors in specific contexts compared to others. That is, the impacts of organizational contextual factors on the newcomers’ proactive behaviors are somehow neglected in research. Consequently, there is gap in the literature on newcomers’ proactive socialization behaviors within the workplace. This should examine the contextual differences that might contribute to newcomers’ proactive behaviors and elucidate the mechanisms through which such behaviors are motivated.

To address these gaps in the literature and elucidate the relationship between newcomers’ proactivity and the work environment, we focused specifically on the role of inclusive leadership—a crucial organizational contextual variable—in promoting such behaviors. This notion is particularly relevant to the generation of newcomers who make up the majority of the workforce because the new generation desires to belong and be recognized by the organization, while also expecting the organization to appreciate their uniqueness and encourage them to excel (Cable et al., 2013; Fang et al., 2019). Inclusive leadership is often assumed to satisfy their demands and lead to favorable modifications in behaviors (Randel et al., 2018). More importantly, we also explain the mechanism of inclusive leadership affecting newcomers’ proactive behaviors. Based on the proactive motivation model (Parker et al., 2010), we examined the mediating role of three proactive motivational states: control beliefs (representing the can-do motivation), state promotion focus (representing the reason-to motivation), and positive affect (representing the energized-to motivation) in the relationship between inclusive leadership and newcomers’ proactive behaviors. In addition, we further examine the moderating effect of newcomers’ power distance orientation (Kirkman et al., 2009) within this process.

**Figure 1:**
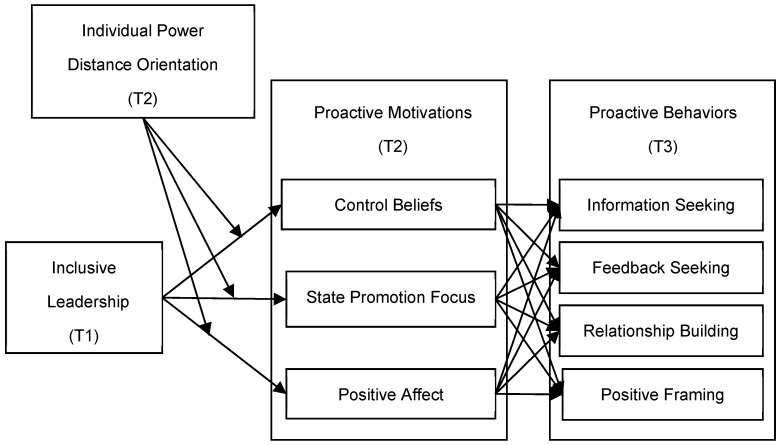
The Conceptual Model.

Through an in-depth examination of how inclusive leadership improves the proactive behavior of newcomers, this study offers robust theoretical support for developing more effective socialization strategies for newcomers within contemporary organizations. This study contributes to the literature in three ways. Firstly, by validating the impact of inclusive leadership on newcomers’ proactive behaviors, we extend the understanding of antecedents to proactive behaviors, which is somewhat scarce in the previous literature (Nguyen et al., 2021). Secondly, this study uncovers the mechanism that influences the proactive socialization behavior of newcomers. This study reveals three proactive motivations underlying newcomers’ decision to proactivity and introduces individual power distance orientation to further clarify the situational conditions under which inclusive leadership influences this process. Thirdly, by empirically testing all three proactive motivational pathways simultaneously, we fully validated Parker et al.’s (2010) theory in the study of organization socialization. The conceptual model is depicted in Figure 1.

## 2. Theoretical Background and Hypotheses Development

### 2.1. Inclusive Leadership

The topic of inclusive leadership is marked by a wide range of perspectives and conceptual frameworks (Korkmaz et al., 2022), yet scholars have not reached a unified understanding of this concept (Randel et al., 2018). The notion was generated by Nembhard and Edmondson (2006) as “Leaders’ languages and behaviors that demonstrate the willingness to engage and value contributions of others”. Subsequent researchers have generally expanded on this definition, conceptualizing it as the demonstration of openness and accessibility in interactions with followers (Carmeli et al., 2010; Hirak et al., 2012). For example, Carmeli et al. (2010) propose that inclusive leadership can be evaluated by its openness, effectiveness, and accessibility in interpersonal communication with subordinates. In a further study, Randel et al. (2018) presented a more extensive comprehension of inclusive leadership, drawing upon the theoretical framework of inclusion as presented by Shore et al. (2011). According to their argument, inclusive leadership refers to the behaviors that simultaneously motivate all team members to perceive themselves as belonging to the workgroup while also encouraging them to contribute their strengths towards achieving positive outcomes for the group.

The study of inclusive leadership by Chinese scholars has started relatively recently, and investigations are still underway. After interviews with managers and employees of 12 Chinese companies with various ownership structures, Tang et al. (2014) revealed that the Chinese perspective on inclusion encompasses a broader range of dimensions compared to the Western understanding. Researchers have found that the idea of inclusion aligns with the Chinese term “Baorong”, encompassing both the Western understanding of the concept and the Chinese aspect of “tolerance”, providing some insights into inclusive leadership studies. According to Fang et al. (2019) the Chinese cultural viewpoint about “inclusiveness” is distinguished by an emphasis on spiritual and moral qualities, such as “tolerance and greatness.” In their study, inclusive leadership in the Chinese workplace is characterized as a set of leadership practices that cover understanding, encouragement, as well as tolerance. These practices are found to more closely align with the psychological needs of the new generation of employees. Consequently, the notion above is incorporated into our study.

### 2.2. Newcomers’ Proactive Behaviors

The proactive behaviors of newcomers can accelerate their adaptation process when entering an organization. It involves self-directed and comprehensive efforts to effect changes in both the individual and the surrounding environment (Ashford & Black, 1996; Bindl & Parker, 2011). Recent studies commonly recognized that newcomers do not just adopt a passive strategy during the socialization process. Instead, they proactively seek out information and purposefully establish social networks (Deng & Yao, 2022). An increasing body of empirical research has provided substantial evidence supporting the association between participating in proactive activities and various favorable outcomes, including enhanced job satisfaction, improved work performance, and reduced turnover intention (Bauer et al., 2019; Cooper-Thomas & Burke, 2012; Saks et al., 2011). Referring to the previous literature (Ashford & Black, 1996; Bauer & Erdogan, 2011), this study examined four types of newcomers’ proactive behaviors, including information seeking, feedback seeking, relationship building, and positive framing.

Initially, Ashford and Black (1996) proposed sensemaking as a crucial behavior for newcomers, which has been categorized as information seeking and feedback seeking in subsequent studies (Cooper-Thomas & Burke, 2012; Kowsikka & James, 2019; Wanberg & Kammeyer-Mueller, 2000). Information seeking refers to newcomers actively acquiring knowledge and comprehending the environment (Wanberg & Kammeyer-Mueller, 2000), which may be achieved by asking questions about their professional responsibilities, such as organizational rules and objectives. Meanwhile, newcomers may face challenges in assessing the adequacy of their performance due to their limited understanding of the organization’s unique context. By proactively feedback seeking, individuals can modify their behaviors in order to fit the company’s culture and expectations better. The frequency of both information seeking and feedback seeking is linked to crucial work attitudes and behaviors (Bauer & Erdogan, 2011; Wanberg & Kammeyer-Mueller, 2000).

Additionally, newcomers may also engage in relationship building as a type of proactive behavior. The term pertains to the behaviors of newcomers who initiate social interactions in the workplace and is considered important in mitigating loneliness and social isolation (Wanberg & Kammeyer-Mueller, 2000). Studies have supported the significance of fostering interpersonal connections with supervisors and colleagues in yielding favorable outcomes in socialization processes (Bauer et al., 2019; Zheng et al., 2021). Lastly, positive framing was introduced by Ashford and Black (1996) as a proactive strategy that individuals often utilize throughout their transition into a new job. It is defined as a cognitive self-management method to “alter their understanding of a situation by explicitly controlling the cognitive frame they place on the situation”. Employees who engage in positive framing may interpret events as supportive rather than aggressive (Kowsikka & James, 2019). This cognitive orientation has been found to be associated with several positive outcomes, including social integration, work satisfaction, innovation, and job performance (Bauer et al., 2019; Boulamatsi et al., 2021; Harrison et al., 2011).

### 2.3. Inclusive Leadership on Newcomers’ Proactive Behaviors

Facilitating the integration of newcomers into the organization constitutes a critical leadership function (Bauer et al., 2007; Bauer & Erdogan, 2011). The proactive behaviors of newcomers are closely related to the leadership style. For example, Wu and Parker (2017) suggest that support from leaders encourages employees to behave proactively in their work. In addition, Wu et al. (2023) state that humble leadership can impact employee proactive behaviors. Similarly to proactive leadership strategies, inclusive leadership can generate favorable outcomes.

First of all, inclusive leaders possess the ability to understand and empathize with newcomers, demonstrating a willingness to listen to new perspectives and accommodate their thoughts and behaviors. This approach enhances newcomers’ sense of belongingness and identification with the organization. Motivated by this positive reinforcement, employees are more likely to be proactively integrated into the organization. Secondly, inclusive leadership emphasizes employee development. Leaders are readily available to provide support and guidance during the socialization process of newcomers, fostering a continuously evolving awareness and promoting proactive behaviors. Finally, inclusive leaders not only accommodate the mistakes of newcomers in the workplace, but also empower them to address tasks and emergencies more courageously without the fear of failure. This fosters an environment where employees are encouraged to take initiative without undue concern. In light of the aforementioned reasoning, we formulate the following hypothesis:

**Hypothesis** **1:** *Inclusive leadership positively predicted newcomers’ proactive behaviors. To be specific, (a) information seeking, (b) feedback seeking, (c) relationship building, and (d) positive framing.*

### 2.4. The Proactive Motivation Model

The proactive motivation model, as proposed by Parker et al. (2010), is usually applied to examine the underlying factors that drive employees to engage in risky or challenging behaviors, such as voice behaviors (Liang et al., 2022; Ng et al., 2021). In their model, leadership, as a contextual factor, can influence employees’ proactive behaviors through three motivational states, including can-do motivation, reason-to motivation, and energized-to motivation (Wu & Parker, 2017). The three aforementioned motivational states are introduced in our study to investigate the impacts of inclusive leadership on newcomers’ proactive behaviors.

First, the motivational state known as can-do motivation encompasses an individual’s conviction to participate in proactive behaviors effectively (Parker et al., 2010). Engaging in proactive behaviors for a newcomer likely involves a deliberate decision process because it usually entails a high level of risk (Ashford & Black, 1996; Cooper-Thomas & Burke, 2012). Given newcomers’ vulnerable and uncertain nature (Peltokorpi et al., 2022), they will likely evaluate their capabilities and the possible consequences before engaging in such behaviors. Proactivity is possible when they feel they can control the situation and have an impact on the outcomes (Frese & Fay, 2001). The control beliefs can represent such a situation, which refers to an individual’s beliefs in that capacity to create a meaningful impact and prevent undesired outcomes (Ng et al., 2021). Thus, an individual’s control beliefs are chosen for this study to indicate the can-do motivation.

Second, the reason-to pathway is another significant mechanism that fosters proactivity, which focuses on compelling reasons for an individual to engage in proactive behaviors (Parker et al., 2010), mostly centered around questions such as “Do I want to do this?” or “Why should I do this?”. Such reason-to motivation may be more critical than can-do motivation in the proactivity process because even individuals with high confidence levels may not exhibit proactive behaviors unless they have compelling reasons to do so (Eccles & Wigfield, 2002). This can-do motivation can be properly represented by an individual’s state promotion focus. Newcomers with a state promotion focus emphasize access to opportunities rather than maintaining the status quo (Neubert et al., 2008), thereby they are more likely to engage in newcomers’ proactive behaviors. Studies have shown that positive leadership can stimulate an individual’s state promotion focus (Wang et al., 2022). Thus, this study will introduce state promotion focus as a reason-to motivation, offering insight into the correlation between inclusive leadership and newcomers’ proactive behaviors.

Lastly, alongside the can-do and reason-to motivation, there exists a third pathway for stimulating proactivity known as the energized-to motivation, wherein positive affect plays a vital role. The experience of positive affect has the potential to elicit positive behaviors. This is because positive affect can result in the experience of energy, which subsequently leads to heightened effort in pursuing proactive goals (Wang et al., 2022; Zhang & Inness, 2019). Therefore, positive affect can represent the energized-to motivation to engage in newcomers’ proactive behaviors. This study develops on previous empirical research (Bindl et al., 2012; Ye et al., 2018) to define positive affect as the general affect experienced by newcomers in the workplace rather than focusing on specific emotions at a particular moment. This approach is taken because the examination of positive affect is conducted within the context of inclusive leadership, which is linked to newcomers’ overall perceptions and experiences.

### 2.5. Control Beliefs as the Can-Do Motivation to Engage in Newcomers’ Proactive Behaviors

Drawing on the proactive motivation model, this study suggests that inclusive leadership, regarded as a contextual factor, may positively impact newcomers’ proactive behaviors via their control beliefs. Due to unclarity and uncertainty regarding the organizational environment (Peltokorpi et al., 2022), newcomers may experience a diminished sense of control. However, inclusive leadership makes newcomers perceive that their efforts are valued by genuinely accepting and appreciating their opinions, thereby fostering a perception among newcomers that they have both influence and control (Randel et al., 2018). This line of reasoning aligns with previous studies demonstrating that experiencing influence in the workplace can improve the feeling of control (Boudrias et al., 2014). Additionally, the perception of being treated with respect and equality, which arises when newcomers’ perspectives are acknowledged and incorporated, also significantly bolsters their control beliefs (Ng et al., 2021). As a result, within the interactions between newcomers and inclusive leaders, their control beliefs are enhanced.

Based on the fundamental premise that can-do motivation promotes more positive behaviors, this study posits a correlation between newcomers’ control beliefs and their proactive behaviors. Specifically, newcomers’ control beliefs are positively associated with the perception of the validity of their proactive behaviors. According to Kopelman and Thompson (1976), employees are more likely to be motivated to be proactive if they believe they can influence the work environment effectively. Control beliefs are crucial in shaping newcomers’ perceptions of the work environment as structured, predictable, and surmountable (Bhanji et al., 2016). As a result, newcomers with strong control beliefs think their behaviors can directly influence work outcomes rather than being determined by external and uncontrollable factors (Ng et al., 2006). The resulting psychological benefit will motivate newcomers to participate in proactive behaviors. Consistent with the abovementioned discussion, this study proposes that inclusive leaders enhance newcomers’ proactive behaviors through high levels of can-do motivation and other control beliefs. Thus, hypothesis 2 suggests:

**Hypothesis** **2.1:** *Inclusive leadership positively predicted newcomers’ control beliefs.*

**Hypothesis** **2.2:** *Control beliefs mediate the positive relationship between inclusive leadership and newcomers’ (a) information seeking, (b) feedback seeking, (c) relationship building, and (d) positive framing.*

### 2.6. State Promotion Focus as the Reason-To Motivation to Engage in Newcomers’ Proactive Behaviors

The state promotion focus, as a reason-to motivation, also has the potential to elucidate a positive correlation between inclusive leadership and newcomers’ proactive behaviors. First, Neubert et al. (2008) believe that state promotion focus was triggered when the workplace environment emphasized the need for growth and gain. Inclusive leaders have the ability to actively engage in attentive listening to newcomers while also demonstrating the capacity to acknowledge and appreciate their accomplishments. In addition, they are able to rationally accept and understand the mistakes of newcomers (Fang et al., 2019). Thus, for employees unfamiliar with the work environment and content, inclusive leadership has the potential to enhance self-efficacy, psychological empowerment, and organizational status (Shore & Chung, 2022). Such outcomes may lead to personal growth and then evoke state promotion focus. Meanwhile, inclusive leaders enhance employees’ perceptions of organizational fairness by treating them equally and respecting their suggestions impartially (Lu et al., 2023). Studies have shown that fairness also satisfies employees’ desire for growth needs, which in turn leads to the state promotion of focus (Liang et al., 2022).

The state promotion focus is particularly relevant to newcomers’ proactive behaviors (Ashford et al., 2003; Ashford & Black, 1996). As proactive activities are often accompanied by risk, newcomers may be more cautious when engaging in them. State promotion focus emphasizes personal achievement, growth, and success and involves pursuing goals following an ideal self-directed framework (Akhtar & Lee, 2014). Under the state promotion focus, newcomers prioritize achieving goals as their utmost priority. Consequently, they may demonstrate a high level of concern for goals related to personal growth, thereby ignoring the risks and losses that may be involved. Related evidence has suggested that state promotion focus drives newcomers to obtain helpful information and feedback about their performance (Ashford et al., 2003). Thus, the state promotion focus, coinciding with the reason-to motivations, encourages newcomers to engage in behaviors that can increase the likelihood of success, such as proactive behaviors. Based on the above analysis, the study proposes that:

**Hypothesis** **3.1:** *Inclusive leadership positively predicted newcomers’ state promotion focus.*

**Hypothesis** **3.2:** *State promotion focus mediates the positive relationship between inclusive leadership and newcomers’ (a) information seeking, (b) feedback seeking, (c) relationship building, and (d) positive framing.*

### 2.7. Positive Affect as the Energized-To Motivation to Engage in Newcomers’ Proactive Behaviors

Finally, a third reason why inclusive leadership is more likely to promote newcomers’ proactive behaviors is that it stimulates the energized-to motivation, referring to the emotional reason that leads to proactive goal pursuit (Parker et al., 2010). Positive affect accurately captures this kind of motivation. Inclusive leadership is likely associated with a more substantial positive affect as it can effectively satisfy newcomers’ needs for belongingness and uniqueness (Ng et al., 2021; Randel et al., 2018; Shore et al., 2011). On the one hand, everyone has an inherent need for social belonging, which is the desire to form meaningful and pleasant interpersonal connections. As with positive relational leadership (Carmeli et al., 2010), inclusive leadership allows newcomers to feel part of the organization by supporting them, ensuring justice and equity, and providing opportunities for shared decision-making. On the other hand, uniqueness refers to an individual’s sense of self-worth in effectively managing the external environment. Inclusive leaders promote newcomers’ uniqueness by fully utilizing their unique talents and opinions, proving that they are capable, influential, and valuable members of the organization (Randel et al., 2018). Since need satisfaction strongly promotes positive affect (Ng et al., 2021), this study suggests that inclusive leadership is likely related to newcomers’ positive affect.

Several theoretical frameworks provide evidence of a correlation between positive affect and proactive behaviors. First, Parker et al. (2010) suggested that positive affect is likely to activate one’s behavioral approach system, whereby newcomers tend to notice the positive possibilities of their behavior and evaluate the results positively. Similarly, Seo et al. (2004) argued that positive affect may influence newcomers’ proactivity by shaping their expectations, utility, and process judgments. That is, positive affect leads individuals to make favorable process judgments about the current activities, which is necessary for individuals to persist in the face of obstacles, and therefore, individuals are more inclined to engage in proactive behaviors. Another explanation is that positive affect increases newcomers’ physiological and psychological resources, such as energy and attention. These resources would make them more optimistic about the outcomes of positive behaviors, as well as help to initiate effort and goal-oriented activities, such as proactive behaviors (Fritz & Sonnentag, 2009). The above discussion leads to Hypothesis 4.

**Hypothesis** **4.1:** *Inclusive leadership positively predicted newcomers’ positive affect.*

**Hypothesis** **4.2:** *Positive affect mediates the positive relationship between inclusive leadership and newcomers’ (a) information seeking, (b) feedback seeking, (c) relationship building, and (d) positive framing.*

### 2.8. Moderating Role of Individual Power Distance Orientation

Inclusive leadership can stimulate newcomers’ proactive motivation, therefore leading to proactive socializing behaviors, while personal value differences influence the extent to which newcomers are impacted by their leaders (Kirkman et al., 2009). Individual power distance orientation is defined as the degree to which individuals within an organization perceive the unequal power distribution to be acceptable (Clugston et al., 2000). Kirkman et al. (2009) suggested that it more directly impacts subordinates’ perception, comprehension, and response to various leader behaviors than other personal values. Under the deeply ingrained culture of officialism and hierarchical power structures in China, employees likely have a propensity for high power distance orientation. However, the power distance orientation within a culture might also depend on diverse personal experiences, which is conceptualized as individual power distance orientation (Koós et al., 2022). Especially in the context of Chinese society, characterized by the coexistence of traditional and modern cultural elements, it is observed that the younger generation employees may exhibit more pronounced variations in their power distance orientation. Hence, this study investigates the potential moderating influence of newcomers’ power distance orientation on this association.

Generally, subordinates with a low power distance orientation believe power should be distributed equally within the organization and tend to interact with leaders more equally. They may be more comfortable and delighted with the understanding, appreciation, and tolerance shown by inclusive leaders. Hence, the effect of inclusive leadership on the proactive behaviors of newcomers may be more substantial among individuals who exhibit lower levels of power distance orientation. On the contrary, individuals with a high power distance orientation tend to believe that leaders naturally possess certain privileges and exercise decision-making authority without necessarily consulting their subordinates. Because of their deeply ingrained personal convictions, those subordinates often perceive they cannot influence leaders’ decisions, resulting in a diminished sensitivity towards the inclusive performance of leaders. The inclusive leadership strategy might also be perceived as superfluous, viewed by them as an unnecessary formalization. This conclusion is also supported by Guo et al. (2022)’s study. We therefore arrive at Hypotheses 5.1–5.3.

**Hypothesis** **5.1:** *Individual power distance orientation moderates the positive effect of inclusive leadership on newcomers’ control beliefs. The positive effect is stronger when the power distance orientation is low than when it is high.*

**Hypothesis** **5.2:** *Individual power distance orientation moderates the positive effect of inclusive leadership on newcomers’ state promotion focus. The positive effect is stronger when the power distance orientation is low than when it is high.*

**Hypothesis** **5.3:** *Individual power distance orientation moderates the positive effect of inclusive leadership on newcomers’ positive affect. The positive effect is stronger when the power distance orientation is low than when it is high.*

The value differences among newcomers shape their perception of contextual factors that drive proactivity, influencing their motivation and determining their subsequent behaviors (Guo et al., 2022). In light of this perspective, we posit that newcomers’ power distance orientation moderates the indirect relationship between inclusive leadership and their proactive behaviors via proactive motivations. Specifically, for newcomers who place significance on equal power relations in organizations, proactive motivations provide crucial mechanisms for elucidating why inclusive leadership might exert a more potent influence on their proactive behaviors. As previously discussed, newcomers place a higher value on the inclusiveness of leadership and exhibit greater sensitivity towards inclusive leadership. This heightened awareness fosters enhanced proactive motivations, which subsequently facilitates the emergence of proactive behaviors. Conversely, newcomers with high power distance orientation exhibit lower sensitivity to inclusive leadership, thereby diminishing the subsequent positive outcomes. Therefore, this study obtains Hypotheses 5.4–5.6.

**Hypothesis** **5.4:** *Individual power distance orientation moderates the indirect effect of inclusive leadership on newcomers’ (a) information seeking, (b) feedback seeking, (c) relationship building, and (d) positive framing via control beliefs. The indirect effect is stronger when the power distance orientation is low than when it is high.*

**Hypothesis** **5.5:** *Individual power distance orientation moderates the indirect effect of inclusive leadership on newcomers’ (a) information seeking, (b) feedback seeking, (c) relationship building, and (d) positive framing via state promotion focus. The indirect effect is stronger when the power distance orientation is low than when it is high.*

**Hypothesis** **5.6:** *Individual power distance orientation moderates the indirect effect of inclusive leadership on newcomers’ (a) information seeking, (b) feedback seeking, (c) relationship building, and (d) positive framing via positive affect. The indirect effect is stronger when the power distance orientation is low than when it is high.*

## 3. Research Methods

### 3.1. Samples and Procedures

A multi-wave, multi-source survey was conducted in China to examine the hypothesis above. According to prior studies (Bauer et al., 2007; Takeuchi et al., 2020), employees who have been with their organizations for less than a year are referred to as newcomers. The study protocol was approved by the Institutional Review Board of the university before the initiation of the study and was conducted in strict adherence to the principles outlined in the Declaration of Helsinki. Online data were collected through a third-party platform in China (Credamo) and all the samples were randomly selected to ensure that newcomers came from different regions, industries, and organizations. Participants received an invitation through the platform, which included a brief overview of the study and an informed consent form. We obtained consent electronically before participants completed the survey. Furthermore, to diminish social desirability, the study team reminded participants of the necessity of answering truthfully for the sake of academic study. Respondents who completed the surveys were compensated RMB 5 each time, with an additional RMB 2 for completing all three waves of surveys as an incentive to complete them.

Data were collected from December 2022 to February 2023 and based on graduation time in China, most respondents had been employed for some time and had a deep understanding of their work content and working environment. According to prior research (Kim et al., 2018), a two-week period is sufficient for investigators to refine and advance their perspectives on the investigated variables. Thus, this study employed a three-wave data collection procedure to eliminate potential biases resulting from common techniques and participant fatigue, with each wave spaced over two weeks (Podsakoff et al., 2003). Our research team gave 450 questionnaires to newcomers in Time 1 and requested them to fill out a survey regarding demographic information and inclusive leadership. Then, 429 newcomers who finished the surveys in Time 1 were questioned again in Time 2 on control beliefs, state promotion focus, positive affect, and individual power distance orientation. In Time 3, 365 newcomers who replied in the first two rounds of the survey were questioned about their proactive behaviors, such as information seeking, feedback seeking, relationship building, and positive framing.

The final sample comprised 353 valid questionnaires, resulting in an overall response rate of 78.44%. Results indicated that out of the total sample size, 192 (54.39%) were women, while 161 (45.61%) were men. The average age was 25.24 years (SD = 10.71). 147 (41.64%) of the newcomers had been in paid employment for less than six months, with the remaining 206 (58.36%) having been in paid work for more than six months but less than 12 months. They came from a variety of industries, with 194 (54.96%) from private companies and 159 (45.04%) in technical jobs. The results were presented in Appendix A.

At the same time, attrition analysis was conducted on the demographic characteristics of the three groups of participants (those who completed the survey, those who dropped out at Time 2, and those who dropped out at Time 3). According to previous studies (Koós et al., 2022), this study adopted a chi-square test to analyze categorical variables, and a non-parametric test (independent sample Kruskal–Wallis test) to analyze continuous variables. Only firm type was found to differ significantly between the sample that dropped out of the study and the sample that completed the study. However, the effect size remained relatively small, suggesting that the difference can be negligible. The results were presented in Appendix B.

### 3.2. Measures

For all variables used in this study, participants were asked to respond on a 7-point Likert scale, with 1 representing “strongly disagree” and 7 representing “strongly agree”. The scales were originally in English and employed a repeated forward–backward translation procedure. The scales were first translated into Chinese, and then the Chinese version was translated into English and compared with the original English version. After that, certain words or phrases in the Chinese version that did not precisely correspond to those in the English version were modified. The measurement items are presented in Appendix C.

Inclusive leadership was measured by an eleven-item scale developed by Fang et al. (2019). A sample item was “When something went wrong, and the leaders did not arbitrarily blame us without understanding the details” (α = 0.913).

This study selected four specific newcomers’ proactive socialization behaviors from Ashford and Black’s (1996) Proactive Behavior Scale. Information seeking and feedback seeking were each measured using four items. Relationship building and positive framing were measured with three items each. Sample items included: “I tried to learn the important policies and procedures in the organization” (information seeking, α = 0.831), “I sought out feedback on my performance during assignments” (feedback seeking, α = 0.905), “I tried to form a good relationship with my supervisor” (relationship building, α = 0.830), and “I tried to look on the bright side of things” (positive framing, α = 0.817).

Respondents’ control beliefs were measured using Mittal and Griskevicius’s (2014) four-item scale (α = 0.860). An example was, “When I really want to do something, I usually find a way to succeed at it”.

A nine-item Work Regulatory Focus Scale (Neubert et al., 2008) was used to measure state promotion focus. Referring to the research of Wang et al. (2022), we adapted the items to assess promotion focus as a state rather than a trait. One example was, “Right now, I take chances at work to maximize my goals for advancement” (α = 0.904).

Positive affect was measured with Watson et al.’s (1988) ten-item Positive and Negative Affect Scale (PANAS) (Watson et al., 1988). The assessed emotions encompassed “enthusiasm, excitement, and interest” (α = 0.937).

A six-item scale developed by Dorfman and Howell (1988) was selected for the power distance orientation (Dorfman & Howell, 1988). This scale was more suitable for the Chinese research context because it was based on a sample of Taiwanese employees in China. An example was “It is frequently necessary for a manager to use authority and power when dealing with subordinates” (α = 0.846).

Based on previous studies (Cooper-Thomas et al., 2014), we controlled participants’ demographic variables, including gender, age, education, work tenure, industry, type of enterprise, and job function. Previous research has indicated a correlation between these variables and newcomers’ proactive behaviors.

## 4. Results

### 4.1. Confirmatory Factor Analysis

Confirmatory factor analyses (CFA) were conducted to compare the hypothetical model with a series of other alternative models. As shown in Table 1, the primary nine-factor model exhibited good fit statistics (χ^2^ = 2245.476, df = 1341, RMSEA = 0.044, SRMR = 0.049, CFI = 0.919, TLI = 0.914). In addition, the model was superior to other models with fewer factors. Therefore, these findings revealed that the proposed model fits the data well and demonstrated the discriminant validity of the focal variables.

### 4.2. Descriptive Statistics

Table 2 illustrated the mean values, standard deviations, and correlation analysis of the major variables. The result showed that inclusive leadership was positively associated with newcomers’ four proactive behaviors: information seeking (r = 0.399, *p* < 0.01), feedback seeking (r = 0.432, *p* < 0.01), relationship building (r = 0.529, *p* < 0.01), and positive framing (r = 0.488, *p* < 0.01). The correlations between inclusive leadership and three proposed mediating mechanisms were also significant: control beliefs (r = 0.568, *p* < 0.01), state promotion focus (r = 0.540, *p* < 0.01), and positive affect (r = 0.488, *p* < 0.01). Furthermore, control beliefs were positively associated with newcomers’ information seeking (r = 0.362, *p* < 0.01), feedback seeking (r = 0.513, *p* < 0.01), relationship building (r = 0.557, *p* < 0.01), and positive framing (r = 0.397, *p* < 0.01). State promotion focus was positively associated with newcomers’ information seeking (r = 0.477, *p* < 0.01), feedback seeking (r = 0.507, *p* < 0.01), relationship building (r = 0.529, *p* < 0.01), and positive framing (r = 0.443, *p* < 0.01). Positive affect was positively associated with newcomers’ information seeking (r = 0.402, *p* < 0.01), feedback seeking (r = 0.561, *p* < 0.01), relationship building (r = 0.508, *p* < 0.01), and positive framing (r = 0.449, *p* < 0.01). Thus, the hypotheses were initially supported by data.

### 4.3. Hypotheses Testing

#### 4.3.1. Tests on the Effects of Inclusive Leadership on Newcomers’ Proactive Behaviors

Regression analysis was conducted to confirm the impact of inclusive leadership on newcomers’ proactive behaviors. After controlling newcomer demographic factors, Table 3 indicated that inclusive leadership significantly and positively predicted newcomers’ information seeking (B = 0.405, *p* < 0.001, Model 1), feedback seeking (B = 0.584, *p* < 0.001, Model 2), relationship building (B = 0.620, *p* < 0.001, Model 3), and positive framing (B = 0.542, *p* < 0.001, Model 4). Thus, Hypothesis 1 was supported by data.

#### 4.3.2. Tests on the Mediation Effects

Firstly, this study verified the impact of inclusive leadership on newcomers’ proactive motivations. Table 4 indicated that inclusive leadership had a positive impact on control beliefs (B = 0.653, *p* < 0.001, Model 5), state promotion focus (B = 0.509, *p* < 0.001, Model 7), and positive affect (B = 0.483, *p* < 0.001, Model 9), which supported Hypotheses 2.1, 3.1, and 4.1.

In addition, to examine the mediating roles of promotion focus, control beliefs, and positive affect, the multiple mediations of the model based on 5000 iterations with a 95% confidence interval were tested by SPSS PROCESS. The results are shown in Table 5. By combining the three mediators, inclusive leadership had indirect effects on newcomers’ information seeking (effect = 0.156, 95% CI = [0.031, 0.273]), feedback seeking (effect = 0.154, 95% CI = [0.021, 0.279]), relationship building (effect = 0.123, 95% CI = [0.043, 0.225]), and positive framing (effect = 0.107, 95% CI = [0.016, 0.215]) through state promotion focus. It also indirectly influenced newcomers’ information seeking (effect = 0.085, 95% CI = [0.012, 0.164]), feedback seeking (effect = 0.170, 95% CI = [0.060, 0.287]), relationship building (effect = 0.114, 95% CI = [0.026, 0.201]), and positive framing (effect = 0.117, 95% CI = [0.028, 0.210]) through positive affect. However, control beliefs mediated only the relationship between inclusive leadership and relationship building (effect = 0.120, 95% CI = [0.007, 0.241]) and were not significant in the other three relationships. As a result, Hypotheses 3.2 and 4.2 are supported, but the data does not fully support Hypothesis 2.2.

This study found that control beliefs played a mediating role in the association between inclusive leadership and relationship building, whereas their significance was not observed in the remaining three relationships. One possible explanation could be attributed to the partial mediation of positive affect in the relationship between control beliefs and positive behaviors, as shown by Ng et al. (2021). To examine this hypothesis, further tests were conducted in this study, and the results were shown in Appendix D. Inclusive leadership indirectly influences newcomers’ four positive behaviors: information seeking (effect = 0.08, 95% CI [0.02, 0.14]), feedback seeking (effect = 0.15, 95% CI [0.06, 0.24]), relationship building (effect = 0.10, 95% CI [0.03, 0.17]), and positive framing (effect = 0.10, 95% CI [0.03, 0.18]) through chain-mediated effects of control beliefs and positive affect. This confirmed that cognitive beliefs about control were closely related to positive affective experiences stemming from such beliefs (Diener et al., 2015).

#### 4.3.3. Tests on the Moderation Effects

This study also tested whether individual power distance orientation moderated the relationship between inclusive leadership and the three proactive motivators. The predictors were mean centered before the interaction was generated. As shown in Table 4, the interaction between inclusive leadership and individual power distance orientation was significantly related to control beliefs (B = −0.179, *p* < 0.01, Model 6), state promotion focus (B = −0.209, *p* < 0.01, Model 7), and positive affect (B = −0.196, *p* < 0.01, Model 8).

**Figure 2:**
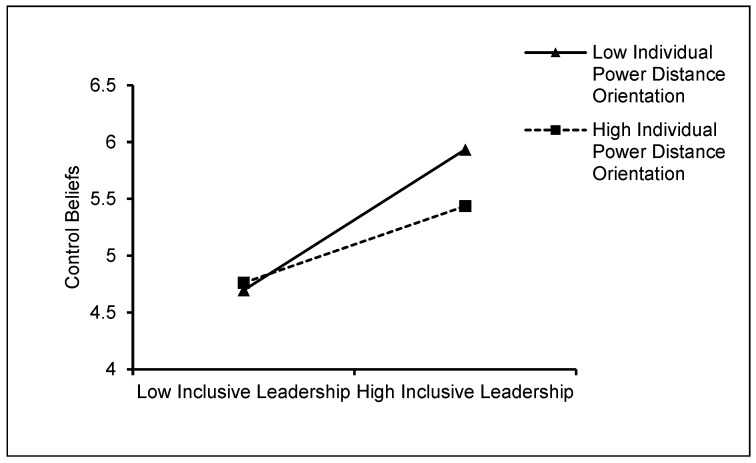
The moderating role of individual power distance orientation of newcomers in the relationship between inclusive leadership and control beliefs.

**Figure 3:**
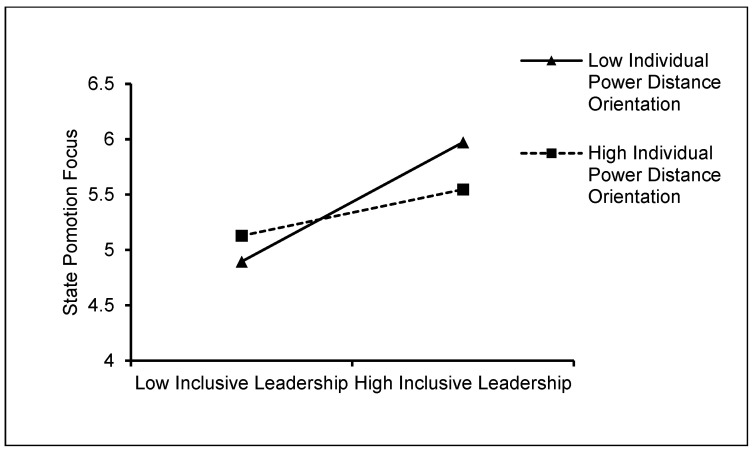
The moderating role of individual power distance orientation of newcomers in the relationship between inclusive leadership and state promotion focus.

**Figure 4:**
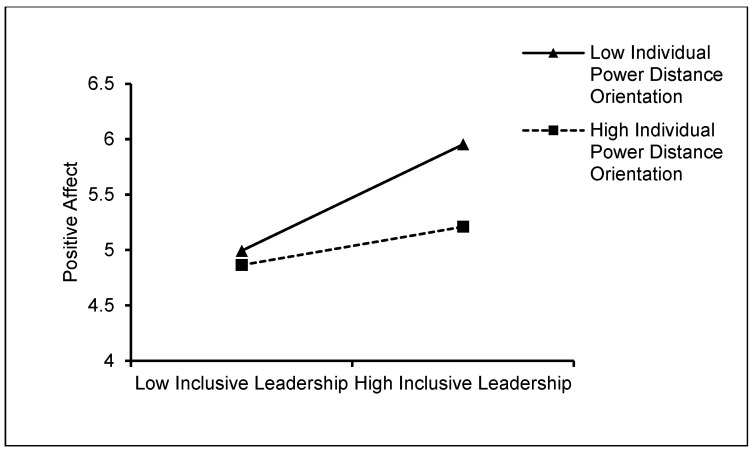
The moderating role of individual power distance orientation of newcomers in the relationship between inclusive leadership and positive affect.

The simple slope graphs for the moderator were displayed at 1SD above and below the mean separately to demonstrate the moderating effect of individual power distance orientation of newcomers. As shown in Figure 2, Figure 3 and Figure 4, the simple slope analysis revealed that when individual power distance orientation was low (1SD below the mean), inclusive leadership had a stronger positive relationship with control beliefs (simple slope = 1.232, *p* < 0.001), state promotion focus (simple slope = 1.078, *p* < 0.001), and positive affect (simple slope = 0.960, *p* < 0.001). Inclusive leadership, however, had weaker relationships with control beliefs (simple slope = 0.678, *p* < 0.001), state promotion focus (simple slope = 0.418, *p* < 0.001), and positive affect (simple slope = 0.345, *p* < 0.001) when their power distance orientation was high (1 SD above the mean). Therefore, Hypotheses 5.1–5.3 were supported.

#### 4.3.4. Tests on the Moderated Mediation Effects

Finally, bias-corrected bootstrapping with the SPSS PROCESS Model 7 was used to investigate the conditional indirect effects. The indirect influence of inclusive leadership on newcomers’ proactive behaviors (information seeking, feedback seeking, relationship building, and positive framing) via state promotion focus and positive affect were stronger at low levels of individual power distance orientation, and the effects were lower or insignificant for high levels of individual power distance orientation. Therefore, the conditional mediating effects of state promotion focus, and positive affect were significant, providing full support for Hypotheses 5.5 and 5.6. Meanwhile, the conditional mediation effect of inclusive leadership influencing newcomers’ proactive behaviors through control beliefs was insignificant, and the data did not support Hypothesis 5.4.

For example, as presented in Table 6, the results of the conditional indirect effects model showed that the indirect influence of inclusive leadership on information seeking via state promotion focus (effect = 0.209, 95% CI [0.038, 0.388]) and positive affect (effect = 0.106, 95% CI [0.014, 0.219]) was stronger among newcomers at low levels of individual power distance orientation. In contrast, at high levels of individual power distance orientation, the indirect influence of inclusive leadership on information seeking via state promotion focus (effect = 0.080, 95% CI [0.024, 0.172]) and via positive affect (effect = 0.037, 95% CI [0.082, 0.162]) were weaker. The index of moderated mediation of state promotion focus (effect = −0.064, 95% CI [−0.127, −0.096]) and positive affect (effect = −0.036, 95% CI [−0.084, −0.003]) were significant. However, the indirect effect of inclusive leadership on information seeking via control beliefs was not significant at low (effect = −0.016, 95% CI [−0.158, 0.122]) and high (effect = −0.008, 95% CI [−0.080, 0.070]) levels of individual power distance orientation. The index of moderated mediation of control beliefs was also insignificant (effect = 0.004, 95% CI [−0.027, 0.043]). Please refer to Appendix D for the full data test results.

## 5. Discussion

The current study provided a better understanding of the organizational socialization process by examining the relationship between inclusive leadership, newcomers’ proactive motivations, their proactive behaviors, and their power distance orientation, drawing on the Chinese cultural ideal of inclusion. The results showed that inclusive leadership positively and indirectly affected newcomers’ proactive behaviors via their reason-to motivation (state promotion focus) and energized-to motivation (positive affect). Furthermore, the positive association between inclusive leadership and two forms of newcomer proactive motives (state promotion focus and positive affect) and their accompanying indirect impacts on newcomers’ proactive behaviors was proved stronger at a low level of individual power distance orientation. However, the mediating effect of the can-do motivation (control beliefs) was not significant, nor was its moderated mediating effect. Previous research on socialization (Ashford & Black, 1996; Ashforth & Saks, 2000) reported that newcomers with temporary control loss may also be motivated to engage in proactive behaviors to regain control. As a result, the effect of control beliefs on proactive behaviors may sometimes be weak. Another explanation proposed by Ng et al. (2021) in their study on voice research was that control beliefs may exert their influence, at least in part, through the mediation of positive affect, and this result was verified in the study.

### 5.1. Theoretical Implications

This work provides several theoretical insights that might inform future studies. By investigating the positive influence of inclusive leadership on newcomers’ proactive behaviors, this study addresses a gap in the literature concerning antecedents of newcomers’ proactive behaviors. As mentioned earlier, research predominantly centers on the potential outcomes of newcomers’ proactive behaviors, yet there is limited discussion regarding the factors that may facilitate such proactivity (Nguyen et al., 2021). This study examines how newcomers’ perception of inclusive leadership influences their proactive behaviors, providing theoretical and empirical support for understanding the antecedents of these behaviors. Thus, it advances the literature on the organizational socialization of newcomers.

Secondly, by elucidating the proximal psychological factors, this study offers a supplementary perspective to existing literature and enhances our comprehension of the psychological mechanisms through which newcomers exhibit proactive behaviors. Even though research has proven that proactive motivation drives proactive behaviors (Parker et al., 2010), it is noteworthy that there has been little incorporation of specific motivation, particularly in organizational socialization research. Based on the proactive motivation model, this study investigates the mediating role of three specific motivations in the relationship between inclusive leadership and newcomers’ proactive behaviors. This study enhances our comprehension of the mechanism through which inclusive leadership influences newcomers’ proactive behaviors. At the same time, it also promotes the application of the proactive motivation model in the research of newcomer socialization.

This study also contributes to the applicability of inclusive leadership in the context of the Chinese workplace. According to Korkmaz et al. (2022), the effect of Chinese cultural values on employees’ understanding of and attitude toward inclusive leadership raises concerns over the applicability of Western theory in guiding research. This study finds that the effect of inclusive leadership on newcomers’ proactive behaviors is higher when newcomers have a low individual power distance orientation. By introducing personal values, a variable that is highly influenced by the current Chinese society and culture, the relationship between the independent and dependent variables is more accurately elucidated. Thus, the contextual conditions for the influence mechanism of inclusive leadership are further expanded.

### 5.2. Managerial Implications

The study’s findings will have an impact on how human resource management and leadership styles are developed in contemporary times. Firstly, this study found that inclusive leaders play an important role in the proactive behaviors of newcomers. Therefore, leaders must cultivate inclusive leadership skills, including being people-oriented, practicing active listening, communicating with empathy, genuinely caring for employees, embracing behaviors and ideas that may diverge from traditional norms, and demonstrating patience and understanding towards newcomers who make mistakes. At the same time, given that managers may encounter many obstacles in their efforts to promote inclusion (Shore & Chung, 2022), the HR department should enhance its collaboration with managers and assist them in cultivating the requisite skills and behaviors to embody the various dimensions of inclusive leadership effectively, and to facilitate the organizational socialization process of newcomers. For example, training leaders on how to express their inclusiveness to newcomers and establishing regular communication mechanisms to help leaders exchange experiences (Korkmaz et al., 2022).

In addition, newcomers need motivation to engage in proactive behaviors. Considering the mediating role of state promotion focus and the positive affect between inclusive leadership and newcomer proactive behaviors, leaders can also provide targeted management strategies from these two perspectives. For instance, to cultivate the state promotion focus, leaders should show that they value the development of newcomers and should be understanding and tolerant of newcomers who make mistakes. This encourages newcomers to gain personal growth and achievement through proactive behaviors, rather than focusing only on avoiding potential losses. Another example is the development of the work environment. Leaders can address their desire for belongingness and uniqueness by encouraging and helping newcomers to contribute fully by soliciting their opinions extensively. This helps to enhance the pleasant and active feelings of the newcomers, which in turn leads to positive affect.

Finally, the results imply that in a collectivist culture such as China, subordinates with low power distance orientation are more susceptible to the impacts of inclusive leadership. Thus, in the workplace, leaders should excel in observing the personality of their subordinates and discerning their power distance orientation. Instead of employing a uniform approach toward all followers, leaders may need to adopt different behaviors while guiding newcomers with varying levels of power distance orientation. For example, for newcomers with low power distance orientation, inclusive leadership may be a good way to increase their motivation and following behaviors. However, for newcomers with high power distance orientation, it may be more appropriate for leaders to engage in paternalistic and authoritative behaviors to facilitate proactivity, given that employees with high power distance orientation prefer their leaders to give explicit and specific directions and requirements.

### 5.3. Limitations and Future Research

In addition, our research has some limitations that provide opportunities for further research. Initially, the variables used in our study were based on self-reported data of newcomers. Consequently, it is crucial to recognize that certain connections identified in our research may be susceptible to common methodological bias. This study uses several procedures recommended by Podsakoff et al. (2003) to address this concern. These procedures included using a time-interval study design, incorporating distinct questionnaire sections and instructions, and providing assurances regarding the confidentiality of responses. However, it is recommended that future research further addresses this issue by soliciting evaluations of the proactive behavior of focal employees from their leaders or coworkers.

Secondly, this study examined the relationship between inclusive leadership and the proactive socialization behaviors of newcomers by identifying three types of proactive motivations. Notably, control beliefs, which serve as the can-do motivation of newcomers, do not necessarily act as a mediating factor in this relationship. Although this study found in subsequent complementary analyses that the utility of control beliefs may be transmitted through positive affect, the effects of a sense of control on individuals’ behaviors may be complex (Ashforth & Saks, 2000). Therefore, future research could conduct a more comprehensive investigation of control beliefs or other mediators more comprehensively to deepen the understanding of newcomers’ positive socialization behaviors.

Finally, this study primarily examines the influence of inclusive leadership, at the individual level, on the proactive behaviors of newcomers, and the possible effects of team- and organizational-level factors are not taken into account. Hence, it is recommended that further studies explore proactive behaviors within the context of teams or organizations because inclusion may occur at various organizational levels (Buengeler et al., 2018). Furthermore, the study did not emphasize the influence of corporate characteristics on individuals’ perceptions of inclusive leadership. Future research may consider conducting a comparative analysis of organizations across various industries, sizes, or stages of development to validate the effectiveness of inclusive leadership more clearly.

## Figures and Tables

**Table 1:** Comparison of Measurement Models for Study Variables.

Models	Descriptions	χ^2^	d.f.	Δχ^2^	RMSEA	SRMR	CFI	TLI
Hypothesize nine-factor model	Inclusive Leadership, Information Seeking, Feedback Seeking, Relationship Building, Positive Framing, Control Beliefs, State Promotion Focus, Positive Affect, Individual Power Distance Orientation	2245.476	1341	-	0.044	0.049	0.919	0.914
Eight-factor model	Information Seeking and Feedback Seeking were integrated into one factor.	2667.695	1349	422.219	0.053	0.061	0.882	0.875
Seven-factor model	Control Beliefs, State Promotion Focus, and Positive Affect were integrated into one factor.	3570.558	1356	902.863	0.068	0.076	0.802	0.791
Six-factor model	Information Seeking and Feedback Seeking were integrated into one factor; Control Beliefs, State Promotion Focus, and Positive Affect were integrated into one factor.	3979.502	1362	408.944	0.074	0.0840	0.766	0.755

**Table 2:** The Descriptions and Correlations of Major Variables.

	Variables	1	2	3	4	5	6	7	8	9
1	Inclusive Leadership									
2	Control Beliefs	0.568 **								
3	State Promotion Focus	0.540 **	0.548 **							
4	Positive Affect	0.401 **	0.593 **	0.489 **						
5	Information Seeking	0.399 **	0.362 **	0.477 **	0.402 **					
6	Feedback Seeking	0.432 **	0.513 **	0.507 **	0.561 **	0.394 **				
7	Relationship Building	0.529 **	0.557 **	0.529 **	0.508 **	0.475 **	0.526 **			
8	Positive Framing	0.488 **	0.397 **	0.443 **	0.449 **	0.474 **	0.364 **	0.456 **		
9	Individual Power Distance Orientation	−0.241 **	−0.265 **	−0.220 **	−0.332 **	−0.196 **	−0.148 **	−0.169 **	−0.188 **	
Mean		5.392	5.261	5.438	5.327	5.636	5.546	5.272	5.433	2.870
SD		0.875	1.044	0.850	1.044	0.881	1.147	1.109	1.013	1.071

** *p* < 0.01.

**Table 3:** Results of Regression Analyses Results Predicting Newcomers’ Proactive Behaviors.

Variables	Information Seeking	Feedback Seeking	Relationship Building	Positive Framing
	Model 1	Model 2	Model 3	Model 4
Gender	0.008	0.007	−0.212	−0.009
Age	−0.003	−0.007	0.041	0.032
Education Level	−0.039	0.011	0.094	−0.033
Organizational tenure	−0.004	0.040	0.028	0.005
Industry	0.028	0.002	0.006	0.008
Type of Enterprise	0.072	0.095	−0.017	0.151
Job Function	−0.088	0.066	0.036	0.084
Inclusive Leadership	0.405 ***	0.584 ***	0.620 ***	0.542 ***
R^2^	0.274	0.306	0.398	0.365
F	9.046	11.146	19.679	15.501

*** *p* < 0.001.

**Table 4:** Results of Regression Analyses Results Predicting Newcomers’ Proactive Motivations.

Variables	Control Beliefs		State Promotion Focus		Positive Affect	
	Model 5	Model 6	Model 7	Model 8	Model 9	Model 10
Gender	−0.046	−0.041	−0.118	−0.131	0.165	0.154
Age	0.024	0.028	0.013	0.017	0.017	0.021
Education Level	−0.082	−0.085	0.104	0.103	−0.027	−0.035
Organizational tenure	0.015	0.017	0.004	0.007	−0.003	−0.002
Industry	0.027	0.038 *	0.003	0.013	−0.015	−0.002
Type of Enterprise	0.027	0.027	0.036	0.036	0.108	0.107
Job Function	0.050	0.060	−0.140	−0.127	0.072	0.075
Inclusive Leadership	0.653 ***	0.628 ***	0.509 ***	0.500 ***	0.483 ***	0.427 ***
Individual Power Distance Orientation		−0.108 *		−0.052		−0.215 ***
Inclusive Leadership * Individual Power Distance Orientation		−0.196 ***		−0.210 ***		−0.205 ***
R^2^	0.325	0.394	0.299	0.386	0.178	0.274
ΔR^2^		0.069		0.097		0.096
F	22.232	22.189	19.803	21.465	9.341	12.910

*** *p* < 0.001. * *p* < 0.05.

**Table 5:** Results of the Indirect Effects.

DV	Model	Effect	SE	Bias-Corrected 95% CI
Information Seeking	Inclusive Leadership → Control Beliefs → Information Seeking	−0.013	0.055	[−0.122, 0.096]
	Inclusive Leadership → State Promotion Focus → Information Seeking	0.156	0.063	[0.031, 0.273]
	Inclusive Leadership → Positive Affect → Information Seeking	0.085	0.039	[0.012, 0.164]
Feedback Seeking	Inclusive Leadership → Control Beliefs → Feedback Seeking	0.112	0.073	[−0.024, 0.262]
	Inclusive Leadership → State Promotion Focus → Feedback Seeking	0.154	0.066	[0.021, 0.279]
	Inclusive Leadership → Positive Affect → Feedback Seeking	0.170	0.057	[0.060, 0.287]
Relationship Building	Inclusive Leadership → Control Beliefs → Relationship Building	0.120	0.059	[0.007, 0.241]
	Inclusive Leadership → State Promotion Focus → Relationship Building	0.123	0.047	[0.043, 0.225]
	Inclusive Leadership → Positive Affect → Relationship Building	0.114	0.046	[0.026, 0.201]
Positive Framing	Inclusive Leadership → Control Beliefs → Positive Framing	−0.029	0.058	[−0.136, 0.09]
	Inclusive Leadership → State Promotion Focus → Positive Framing	0.107	0.051	[0.016, 0.215]
	Inclusive Leadership → Positive Affect → Positive Framing	0.117	0.046	[0.028, 0.210]

**Table 6:** Results of the moderated mediation effect test with information seeking as the dependent variable.

Model	Estimate	SE	Bias-Corrected 95% CI
Effects of inclusive leadership on information seeking via control beliefs			
Low Individual Power Distance Orientation	−0.016	0.070	[−0.158, 0.122]
High Individual Power Distance Orientation	−0.008	0.037	[−0.080, 0.070]
Index of moderated mediation	0.004	0.018	[−0.027, 0.043]
Effects of inclusive leadership on information seeking via state promotion focus			
Low Individual Power Distance Orientation	0.209	0.084	[0.038, 0.388]
High Individual Power Distance Orientation	0.080	0.043	[0.024, 0.172]
Index of moderated mediation	−0.064	0.030	[−0.127, −0.096]
Effects of inclusive leadership on information seeking via positive affect			
Low Individual Power Distance Orientation	0.106	0.050	[0.014, 0.219]
High Individual Power Distance Orientation	0.034	0.029	[0.082, 0.162]
Index of moderated mediation	−0.036	0.021	[−0.084, −0.003]

## Data Availability

The datasets generated during and/or analyzed during the current study are available from the corresponding author upon reasonable request.

## Appendix A. Demographic Characteristics of the Samples

**Variables**		**Frequency** **(*n* = 353)**	**Percentage** **(%)**
Gender	Men	161	45.61
	Women	192	54.39
Age	M (SD)	25.24 (3.27)	
Education level	junior college or less	50	14.16
	undergraduate degrees	252	71.39
	postgraduate education or above	51	14.45
Organizational tenure	M (SD)	7.10 (3.22)	
Industry	Internet	46	13.03
	Real estate	24	6.80
	Construction	37	10.48
	Catering	39	11.05
	Education	42	11.90
	Finance	42	11.90
	Manufacturing	65	18.41
	Medical	8	2.27
	Communication	16	4.53
	Energy	19	5.38
	Transportation	15	4.25
Type of enterprise	State-owned enterprise	125	35.41
	Private enterprise	194	54.96
	Foreign capital enterprise	34	9.63
Type of job	Management	134	37.96
	Technology	159	45.04
	Skills	60	17.00

## Appendix B. Attrition Analysis Results

**Variables**	**Completed the Survey** **(N = 353)** ***n* (%)**	**Dropped-Out at T2** **(N = 64)** ***n* (%)**	**Dropped-Out at T3** **(N = 12)** ***n* (%)**	**χ^2^ Test**	**Cramer’s V**	***p***
Gender				1.28	0.06	0.53
Men	45.6	53.1	50			
Women	54.4	46.9	50			
Education level				9.21	0.10	0.06
junior college or less	14.2	1.6	8.3			
undergraduate degrees	71.4	79.7	66.7			
postgraduate education or above	14.4	18.8	25.0			
Industry				26.97	0.18	0.14
Internet	13.0	6.3	8.3			
Real estate	6.8	7.8	16.7			
Construction	10.5	6.3	8.3			
Catering	11.0	12.5	0.0			
Education	11.9	12.5	8.3			
Finance	11.9	9.4	8.3			
Manufacturing	18.4	14.1	0.0			
Medical	2.3	6.3	16.7			
Communication	4.5	6.3	16.7			
Energy	5.4	10.9	8.3			
Transportation	4.2	7.8	8.3			
Type of enterprise				20.86	0.16	<0.001
State-owned enterprise	35.4	34.4	16.7			
Private enterprise	55.0	40.6	41.7			
Foreign capital enterprise	9.6	25.0	41.7			
Job Function				5.39	0.08	0.25
Management	38.0	29.7	58.3			
Technology	45.0	45.3	33.3			
Skills	17.0	25.0	8.3			
Variables	M (SD); Median	M (SD); Median	M (SD); Median	Kruskal–Wallis test	*df*	*p*
Age	25.24 (3.27); 25	25.67 (3.54); 25	27.50 (3.87); 27.50	2.96	2	0.23
Organizational tenure	7.10 (3.22); 7	7.30 (3.41); 7	7.08 (3.65); 7	0.13	2	0.94
Inclusive leadership (T1)	5.39 (0.88); 5.55	5.20 (0.90); 5.31	5.31 (0.71); 5.50	3.51	2	0.17

## Appendix C

Inclusive Leadership Scale
  Encouragement and Recognition to Employees
  In my work, the leaders actively ask my opinions and thoughts.
    The leaders recognize the contribution of my efforts.
    For my work, the leaders encourage me to come up with plans and ideas.
    The leaders recognize our cooperation and exchanges across departments.
    The leaders openly recognize the achievements of employees.
  Respect and Fair Treatment for Employees
  The leaders treat us equally and always adhere to certain commonly recognized principles.
    The leaders focus on fairness and justice when managing teams.
    The leaders treat employees fairly.
    When employees make mistakes, the leaders express emotional understanding and suggestions for improvement.
    The leaders can rationally accommodate our mistakes.
    When something goes wrong, the leaders do not arbitrarily blame us without understanding the details.
Positive Affect
  During the last 4–5 weeks, to what extent did you feel the following at work?
    Enthusiastic
    Interested
    Determined
    Excited
    Inspired
    Alert
    Active
    Strong
    Proud
    Attentive
Proactive socialization behaviors
  Information Seeking
  I tried to learn the (official) organizational structure.
    I tried to learn the important policies and procedures in the organization.
    I tried to learn the politics of the organization.
    I tried to learn the (unofficial) structure.
  Feedback Seeking
  I sought feedback on my performance after assignments.
    I solicited critiques from my boss.
    I sought out feedback on my performance during assignments.
    I asked my boss’s opinion of my work.
  Build Relationships
  I tried to spend as much time as you could with your boss.
    I tried to form a good relationship with my boss.
    I work hard to get to know my boss.
  Positive Framing
  I tried to see my situation as an opportunity rather than a threat.
    I tried to look on the bright side of things.
    I tried to see my situation as a challenge rather than a problem.
Control Beliefs
  I can do just about anything that I really set my mind to.
    Whatever happens in the future mostly depends on me.
    When I really want to do something, I usually find a way to succeed at it.
    Whether or not I am able to get what I want is in my own hands.
State Promotion Focus
  Right now, I take chances at work to maximize my goals for advancement.
    Right now, I tend to take risks at work in order to achieve success.
    Right now, if I had an opportunity to participate in a high-risk, high-reward project I would definitely take it.
    Right now, if my job did not allow for advancement, I would likely find a new one.
    Right now, A chance to grow is an important factor for me when looking for a job.
    Right now, I focus on accomplishing job tasks that will further my advancement.
    Right now, I spend a great deal of time envisioning how to fulfill my aspirations.
    Right now, my work priorities are impacted by a clear picture of what I aspire to be.
    Right now, I am motivated by my hopes and aspirations.
Individual Power Distance Orientation
  Managers should make most decisions without consulting subordinates.
    It is frequently necessary for a manager to use authority and power when dealing with subordinates.
    Managers should seldom ask for the opinions of employees.
    Managers should seldom ask for the opinions of employees.
    Managers should not delegate important tasks to employees.
    Managers should not delegate important tasks to employees.

## Appendix D

**Table A1:** Results of the Conditional Indirect Effects.

Model	Estimate	SE	Bias-Corrected 95% CI
DV: Information Seeking			
Effects of inclusive leadership on information seeking via control beliefs			
Low Individual Power Distance Orientation	−0.016	0.070	[−0.158, 0.122]
High Individual Power Distance Orientation	−0.008	0.037	[−0.080, 0.070]
Index of moderated mediation	0.004	0.018	[−0.027, 0.043]
Effects of inclusive leadership on information seeking via state promotion focus			
Low Individual Power Distance Orientation	0.209	0.084	[0.038, 0.388]
High Individual Power Distance Orientation	0.080	0.043	[0.024, 0.172]
Index of moderated mediation	−0.064	0.030	[−0.127, −0.096]
Effects of inclusive leadership on information seeking via positive affect			
Low Individual Power Distance Orientation	0.106	0.050	[0.014, 0.219]
High Individual Power Distance Orientation	0.034	0.029	[0.082, 0.162]
Index of moderated mediation	−0.036	0.021	[−0.084, −0.003]
DV: Feedback Seeking			
Effects of inclusive leadership on feedback seeking via control beliefs			
Low Individual Power Distance Orientation	0.136	0.090	[−0.029, 0.320]
High Individual Power Distance Orientation	0.070	0.047	[−0.016, 0.169]
Index of moderated mediation	−0.034	0.027	[−0.095, 0.006]
Effects of inclusive leadership on feedback seeking via state promotion focus			
Low Individual Power Distance Orientation	0.206	0.087	[0.033, 0.372]
High Individual Power Distance Orientation	0.079	0.042	[0.009, 0.172]
Index of moderated mediation	−0.063	0.031	[−0.127, −0.008]
Effects of inclusive leadership on feedback seeking via positive affect			
Low Individual Power Distance Orientation	0.213	0.077	[0.073, 0.390]
High Individual Power Distance Orientation	0.069	0.048	[−0.019, 0.171]
Index of moderated mediation	−0.072	0.040	[−0.166, −0.013]
DV: Relationship Building			
Effects of inclusive leadership on relationship building via control beliefs			
Low Individual Power Distance Orientation	0.147	0.073	[0.007, 0.294]
High Individual Power Distance Orientation	0.075	0.044	[0.004, 0.173]
Index of moderated mediation	−0.036	0.021	[−0.082, 0.002]
Effects of inclusive leadership on relationship building via state promotion focus			
Low Individual Power Distance Orientation	0.165	0.063	[0.055, 0.302]
High Individual Power Distance Orientation	0.063	0.029	[0.016, 0.131]
Index of moderated mediation	−0.051	0.025	[−0.082, 0.002]
Effects of inclusive leadership on relationship building via positive affect			
Low Individual Power Distance Orientation	0.143	0.063	[0.030, 0.277]
High Individual Power Distance Orientation	0.046	0.033	[−0.012, 0.115]
Index of moderated mediation	−0.048	0.029	[−0.119, −0.005]
DV: Positive Framing			
Effects of inclusive leadership on positive framing via control beliefs			
Low Individual Power Distance Orientation	−0.035	0.071	[−0.173, 0.104]
High Individual Power Distance Orientation	−0.018	0.037	[−0.09, 0.063]
Index of moderated mediation	0.019	0.024	[−0.024, 0.051]
Effects of inclusive leadership on positive framing via state promotion focus			
Low Individual Power Distance Orientation	0.144	0.066	[0.023, 0.283]
High Individual Power Distance Orientation	0.055	0.033	[0.006, 0.133]
Index of moderated mediation	−0.044	0.023	[−0.096, −0.006]
Effects of inclusive leadership on positive framing via positive affect			
Low Individual Power Distance Orientation	0.147	0.058	[0.037, 0.262]
High Individual Power Distance Orientation	0.047	0.035	[−0.011. 0.127]
Index of moderated mediation	−0.050	0.027	[−0.112, −0.007]

**Table A2:** The Results of the Chain Mediation Analysis.

Model	Effect	SE	Bias-Corrected 95% CI
Direct effect: Inclusive Leadership → Information Seeking	0.266	0.058	[0.152, 0.381]
Indirect effect:			
Inclusive Leadership → Control Beliefs → Information Seeking	0.026	0.049	[−0.071, 0.124]
Inclusive Leadership → Positive Affect → Information Seeking	0.029	0.021	[−0.004, 0.076]
Inclusive Leadership → Control Beliefs → Positive Affect → Information Seeking	0.084	0.029	[0.029, 0.139]
Total indirect effects	0.140	0.041	[0.064, 0.226]
Total effects:	0.405	0.051	[0.306, 0.505]
Direct effect: Inclusive Leadership → Feedback Seeking	0.237	0.068	[0.104, 0.369]
Indirect effect:			
Inclusive Leadership → Control Beliefs → Feedback Seeking	0.150	0.074	[0.010, 0.300]
Inclusive Leadership → Positive Affect → Feedback Seeking	0.051	0.032	[−0.007, 0.120]
Inclusive Leadership → Control Beliefs → Positive Affect → Feedback Seeking	0.147	0.044	[0.064, 0.236]
Total indirect effects	0.347	0.069	[0.210, 0.484]
Total effects:	0.584	0.065	[0.457, 0.711]
Direct effect: Inclusive Leadership → Relationship Building	0.333	0.063	[0.209, 0.457]
Indirect effect:			
Inclusive Leadership → Control Beliefs → Relationship Building	0.150	0.060	[0.036, 0.271]
Inclusive Leadership → Positive Affect → Relationship Building	0.035	0.024	[−0.004, 0.089]
Inclusive Leadership → Control Beliefs → Positive Affect → Relationship Building	0.101	0.037	[0.032, 0.178]
Total indirect effects	0.286	0.060	[0.173, 0.406]
Total effects:	0.620	0.063	[0.505, 0.734]
Direct effect: Inclusive Leadership → Positive Framing	0.408	0.063	[0.284, 0.531]
Indirect effect:			
Inclusive Leadership → Control Beliefs → Positive Framing	−0.002	0.055	[−0.106, 0.111]
Inclusive Leadership → Positive Affect → Positive Framing	0.035	0.024	[−0.006, 0.091]
Inclusive Leadership → Control Beliefs → Positive Affect → Positive Framing	0.102	0.035	[0.032, 0.173]
Total indirect effects	0.135	0.050	[0.050, 0.246]
Total effects:	0.542	0.055	[0.434, 0.650]
